# Polymer Processing and Surfaces

**DOI:** 10.3390/polym13040536

**Published:** 2021-02-11

**Authors:** Michal Sedlačík

**Affiliations:** 1 Centre of Polymer Systems, University Institute, Tomas Bata University in Zlín, Tr. T. Bati 5678, 76001 Zlín, Czech Republic; msedlacik@utb.cz; 2 Department of Production Engineering, Faculty of Technology, Tomas Bata University in Zlín, Vavreckova 275, 76001 Zlín, Czech Republic

Polymer processing and surfaces are considered key parameters for developing unique materials for various applications. While developing the next generation of polymeric and composite materials, the material’s resources, the utility properties of the product, its economy aspects, as well as environmental issues can be optimized. The surface of the filler or final product is of particular interest with respect to the interaction with its surroundings; hence, its modification can positively affect the stimuli-responsive character, adhesion, absorption, tribological properties or biomedical application, among others [1,2,3,4,5].

This special issue, which consists of 25 articles written by research experts in their topic of interest, reports the most recent research on polymer processing, with an emphasis on surface properties. Several novel and fascinating methods related to polymer fabrication, biointerfaces, surface modification, characterization, sensitivity to the surroundings and applications are introduced.

First, the polymer fabrication conditions affecting the resulting properties were investigated. Zhou and coworkers studied the effect of organic cage compounds, namely cucurbit[6]uril, hemicucurbit[6]uril and *β*-cyclodextrin, acting as nucleating agents in polymer foaming materials (specifically polypropylene in this study) on the final structure of the system, not only through an in situ visual injection molding analysis but also through the classic nucleation theory, all due to the fact that the specific strength and dimensional stability of foaming materials are key parameters of foaming materials in terms of their application [6]. They proved that a natural cavity structure of cucurbit[6] determining the low surface density of the nucleating agent had contributed to the reduction of the energy value of the gas–melt interface, which led to the highest nucleation efficiency.

Lu et al. developed a hybrid processing method for producing microstructured light-emitting diode (LED) diffusion plates based on an interesting combination of microgrinding technology and microinjection molding, which is characterized by high efficiency in terms of mass production and being low-cost [7]. The prepared microstructured diffusion LED plates had an approximately 41% higher illumination when compared to traditional diffusion plates. The improved illumination efficiency was the result of a thorough evaluation of the microinjection molding process parameters and the controlled microgrooved array structures on the surface of a mold core that was made by using high-precision microgrinding technology.

The chemical modification of starch is a current topic in the field of chemical reactions, as it can be advantageously used, for example as a macroinitiator. Li et al. followed this trend in their study by examining the effect of the synthesis process parameters of bromoisobutyryl esterified starch (BBES) on its adhesion-to-fibre and film formation abilities [8]. In this preliminary research, they observed how variations in the amount of 2-bromoisobutyryl bromide (BIBB), a catalyst represented by 4-dimethylaminopyridine, the reaction temperature and time affected the bonding forces of the sized polylactic acid (PLA) and polyester roving. The bonding forces followed a peak dependence with an increasing degree of substitution of BBES confirming a positive effect of introducing 2-bromobutyryl substituents into the starch molecules via the bromoisobutyryl esterification of acid-converted starch with BIBB, as the adhesion of BBES to PLA and the polyester fibers were improved while the film brittleness was reduced.

Li and coworkers investigated the effect of four types of radiation sources represented by electron/proton/carbon ion beams on the electrical properties, the mechanical properties and the thermal properties of polyimide (PI) [9]. The most significant difference between the original and the irradiated PI films was observed in the electrical properties. The dielectric constant of the PI films decreased with increasing irradiation fluences, while the dielectric loss of the irradiated PI films increased with a critical frequency increase to 10^4^ Hz rather than with the different fluences that had an effect on the dielectric constant. The described changes were caused by the degradation of the polytetrachloric group present in the PI, manifested by an increased content of nonpolar groups, such as C–C bonds, after irradiation.

The superhydrophobic modification of a water-borne UV lacquer product performed by Wu et al. is also of fundamental interest, as such a modification can make the final product have antifouling, self-cleaning properties as well as an improved resistance to water and erosion [10]. An easy and environmentally friendly way to obtain the aforementioned advantages was constructed while preparing a water-based UV-cured wood-coating modified with the addition of a zinc oxide (ZnO) additive and a stearic acid surface modifier. Here, the nonpolar long chain of the alkyl group of the stearic acid vapor molecule reacted under acidic conditions with the hydroxyl group in acetic acid and caused the displacement of the ZnO metal ions to the stearic acid. This resulted in the generation of a globular zinc stearate, which was grafted together with hydrophobic groups –CH_3_, resulting in the construction of a micro-/nanolevel multistage flower zinc stearate coarse structure. Finally, a good resistance of the as-prepared coating on poplar wood to an acid medium and some organic solvent corrosion ability was demonstrated in this study.

Fiber-reinforced polymer (FRP) composites are very popular in many applications due to their properties. Surprisingly, however, there is a lack of experimental studies of FRP composites with a high concentration of natural fibers, usually with a volume fraction higher than 50%, which can retain good mechanical properties and are very attractive from an environmental point of view. This lack of relevant studies is partially eliminated by the work of Karvanis and coworkers, in which the authors looked at the thermal analysis and mechanical properties of basalt FRP composites with an epoxy matrix [11]. Composites having 20 layers of basalt fibers in twill 2/2 weaves were prepared by a manual laying via compression molding. This experimental study confirmed the high strength-to-weight and high stiffness-to-weight characteristics of the basalt FRP composite due to a very good interfacial bond between the epoxy matrix and the basalt fibers, which together with the high thermal resistance of the basalt fibers makes the composites an ideal candidate for the areas surrounding the exhaust systems of airplanes, cars and vehicles.

In their study, Feng et al. prepared mullite multineedle whiskers connected to one center via a pressure field-assisted polycondensation nonaqueous precipitation method [12]. These particles could be used as a filler in epoxy resin, as the mechanical properties could be significantly improved due to their unique shape, as the whiskers had an aspect ratio of >15 (100 nm in diameter). The polycondensation reaction between anhydrous aluminium fluoride and tetraethyl orthosilicate was optimized in this study in order to achieve high yields.

An excellent protective efficiency in the corrosion applications of various metal substrates is very important for coating manufacturers. Rodošek et al. contributed to the field of epoxy coatings by developing a protective coating with a shorter drying time and shorter minimum overcoat intervals, allowing higher line speeds, which in turn led to a reduction in energy consumption in their study [13]. Octaglycidyl-polyhedral oligomeric silsesquioxane was used as a modifier for the diglycidyl ether of bisphenol-A epoxy resin, which was prepared by glycidyl ring-opening polymerization using dicyandiamide as a curing agent. The authors also used three different accelerators for crosslinking and observed not only their contribution to epoxy ring-opening but also the crosslinking itself when 2-methylimidazole and commercial Curezol 2MZ-A were used, which overall reduced the curing operating temperature and the surface roughness, while the final protective coating was more homogeneous.

In their work, Otaegi at al. concentrated on increasing the mechanical and electrical properties of commercial bio-based polyamide 4,10 by adding two different types of multiwalled carbon nanotubes (CNTs) [14]. They investigated the effect of CNTs’ properties, such as the aspect ratio, and the method for preparing composite materials, using CNTs in powder form or a PA6-based masterbatch, which led to different degrees of dispersion on the said properties. Using Nanocyl CNTs, it was shown that much better electrical and mechanical properties were obtained due to a better dispersion and a higher aspect ratio in the final biobased composite when compared with Cheap Tubes CNTs, which were shortened in length during the melt processing due to the applied shear stress. Furthermore, CNTs caused a much more significant reinforcing effect when compared to the standard polyamide 4,10/CNTs composite when a ternary composite with another PA6 was investigated.

The second part of this special issue deals with biointerfaces, which represent the area of contact between various materials that are considered to be living with another material, representing the theme of ever-increasing demand. This part begins with a study by Swar and coworkers who combined the antifouling and antibacterial properties of Poly(ethylene glycol) (PEG), useful in the biomedical industry, with Nylon 6, a polyamide often used for biomedical textiles [15]. Because the antifouling activity of PEG is related to the polymer chain length, the authors used a conjugation technique based on the immobilization of longer PEG chains on the reduced Nylon 6 surface through their covalent attachment via *N*,*N*’-disuccinimidyl carbonate. The desired surface immobilization was supported by in vitro cytocompatibility experiments and bacterial adhesion testing based on *S. aureus* and *P. Auruginosa*.

The adsorption of bacteria and microorganisms in wastewater treatment is another important feature of biointerfaces. Hence, Cai et al. prepared a poly(vinyl chloride) (PVC) biofilm by blending PVC with poly(vinyl alcohol) (PVA) and cationic polyacrylamide (cPAM) [16]. In addition, the surface area of the PVC biofilm was increased by azodicarbonamide modified with ZnO (mAC). The analysis of the PVC biofilm revealed a significant improvement in hydrophobicity, expressed in terms of the contact angle, as the effect of hydroxyl and amino groups contained in PVA and cPAM, respectively. The zeta potential of the PVC biofilm changed from negative (pure PVC) to positive, which was more conducive to bacteria adsorption. The addition of mAC eventually contributed to an increase in the surface area, which also contributed to an improved hydrophobicity and electrophilicity.

The next part of the special issue concerns the surface modification of polymers from different points of view. The first study in this section, presented by Gim et al., seeks to eliminate the gloss transition defect on high-gloss injection-molded surfaces [17]. The mitigation of this phenomenon has practical reasons, as it creates a bad impression of the product’s visual quality. The authors observed the occurrence of the gloss transition under different molding conditions in detail. They concluded that the key parameters affecting surface gloss were the mold temperature and flow front speed, so to eliminate the gloss transition defect, the nonuniformity of the mold temperature and fluctuation in the flow front speed should be minimized.

Properly applying statistical methodologies can reduce many random attempts, saving not only time and expense but often also benefiting environmental aspects. In their study, Rezic and Kis used the Design of Experiment (DoE) methodology to obtain the optimal parameters for polymer surface functionalization [18]. They used three different fabric samples and enhanced their hydrophobicity to improve their antimicrobial properties by using two different hydrophobic reagents. Six process parameters, which were the fabric weight, dye concentration, hydrophobic reagent conditions, temperature, pressure and particular types of hydrophobic reagents in 42 preliminary experiments, were involved in the DoE and found to be the optimal ones.

Variable modified cellulose is a current topic of high-voltage direct current power transformers. Li and coworkers increased the performance of the insulation pressboard in terms of an improved charge injection inhibition and hydrophobic properties, while the other basic properties were not impaired [19]. They made a surface-treated cellulose insulation pressboard with nanostructured ZnO and polytetrafluoroethylene (PTFE) via reactive radio frequency magnetron sputtering. Here, as in the previously described study, the surface modification process was predicted using a simulation of the interaction between the mentioned surface modifiers in Accelrys Materials Studio, which showed a better adhesion strength of ZnO nanoparticles with cellulose molecules, clearly indicating the sequence of the given materials during sputtering. Using the procedure described above, an approximately 40% reduction in the amount of accumulated space charge and an increase in the water contact angle of 116° was observed for the cellulose pressboard sputtered with ZnO/PTFE when compared to an unmodified cellulose insulation pressboard.

As a topic of this subsection, surface treatment is also important in fine surgery, during which the low friction properties of surgical sutures are important in preventing tissue damage. In their study, Zhang et al. prepared a biological coating, which consisted of dopamine hydrochloride and graphene oxide, on the surface of multifilament surgical sutures [20]. Among other tests, they compared the coefficients of friction of the examined surgical sutures penetrating through the skin substrate, with the result that the modification did not affect this property. However, the modification improved the wettability of the surface, which is a promising result for the required low-injury surgery methods.

Poly(vinyl chloride) is a widely used polymer, as described for example in [16]. Further research on PVC was conducted by Mohammed et al., who developed PVC films with an improved photodegradation stability [21]. They prepared a number of different tin complexes, which differed in chemical structure, and used them as photostabilizers for PVC films. The presence of these tin complexes in PVC films significantly reduced undesirable changes in the polyene and carbonyl functional group indices and the molecular weight of the samples exposed to a defined UV radiation. All tin complexes directly absorbed UV irradiation and acted as primary and secondary stabilizers, preferentially absorbing irradiation with a subsequent slow release of heat energy into the PVC chain over time at a harmless level. The highest efficiency was observed for tin complexes with a high aromaticity.

In industrial conditions, the surface modification of polymeric substrates must be as effective as possible. Atmospheric pressure plasma jets produce a highly reactive chemistry under near-ambient temperature conditions, which is important for treating polymeric packaging, thin film deposition or biomedical applications. However, the use of this technique is sometimes very limited due to the treatment of complex shapes such as hollow objects, tubes or large and irregular surfaces. While treating a long flexible polyethylene terephthalate tube, Nishime et al. focused on the influence of two important processing parameters, namely the use of a grounded substrate holder and different jet incidence angles, on the resulting modification areas [22]. Larger treated zones with an increased treatment homogeneity were achieved when the plasma jet was applied parallel to the surface, which was confirmed by three independent methods, namely the water contact angle mapping method, the distribution of reactive oxygen species expanding on the starch-iodine-agar plates and the UV-VIS irradiation profiles’ analysis.

This special issue also contains one article in which, instead of processing polymers or their surface-related aspects, a surface characterization using a technique that is not very common is employed. Kakani and coworkers used the inverse gas chromatography technique to characterize the surface thermodynamics of the Poly(vinylidene chloride-co-acrylonitrile) copolymer in terms of its London dispersive surface energy, Gibbs free energy and Guttmann Lewis acid-base parameters [23]. In addition, they also observed the surface parameters of this copolymer, such as texture/roughness, intricate patterns, directionality and particle area distribution among others, using computer vision and an image processing analysis of scanning electron microscopy images. The main results of the study were that the London dispersive surface free energy decreased linearly with an increasing temperature and that the investigated copolymer contained more basic sites when than acidic ones, which led to an easier association in an acidic environment.

The final part of this special issue deals with the sensitivity of polymers to their surroundings and the importance of the surface of polymers for various applications. Slobodian and coworkers continued their interesting research on free-standing nanopaper, consisting of a porous network of multiwalled carbon nanotubes, by embedding it into a thermosetting epoxy resin [24]. This strategy has led to several advantages of the prepared composite. Firstly, it was possible to better monitor the curing process of the epoxy resin by means of Joule heating regulation. The presence of carbon nanotube nanopaper in the composite also accelerated its modeling into various shapes when heated by Joule heating and also contributed to its better shape recovery process. The electrically conductive filler also made it possible to detect changes expressed in terms of electrical resistance during the tensile deformation of the composite. Finally, when the composite was an adhesive between two steel strips, the bond strength could be reversibly changed by heating the system to above the glass transition temperature.

Another adhesive system representing a pressure-sensitive adhesive with balanced adhesive and cohesive properties was prepared by Fuensanta et al. by blending two thermoplastic polyurethanes (TPUs), which had very different individual properties [25]. The authors used two different blending procedures, namely separately blending methyl ethyl ketone (MEK) solutions with the two TPUs and blending TPUs in MEK simultaneously. The blending process affected the viscoelastic properties of the TPU blends, which can be crucial in industrial processing. Finally, an optimal combination of the TPUs involved in this study was obtained in terms of a good tack, high 180° peel strength and sufficient cohesion.

An increased resistance to moisture absorption is highly desirable when using polyamide 6 (PA6) in a product, as this polymer undergoes rapid degradation by the hydrolysis of the amide bond under humid conditions, resulting in a lowered durability. This is often supressed by the addition of high-concentration nanotubular fillers, but this could reduce the desired properties, and the additive/PA6 interface is a privileged place for moisture accumulation. Thus, Sabatini and her team investigated the improved resistance of PA6 to hydrothermal ageing attained by dispersing only a very small number of halloysite nanotubes (HNT) in the system [26]. They evaluated the effect of various parameters, such as differently sourced HNT differing in aspect ratios, its amino-silanization with (3-aminopropyl)triethoxysilane or dispersion techniques covered by in situ polymerization or melt blending, on the extended system’s durability. The incorporation of only a very small amount of HNT additive led to many advantages, such as a lower polymer appearance change, a less extended filler/polymer interface reducing the possible moisture accumulation, an unaffected growth of homogeneous PA6 crystals and a generally improved final composite performance regardless of the parameters used. To highlight some results, the in situ polymerization of silane-functionalized HNT resulted in a closed and well-penetrated filler within the PA6 matrix, leading to a significantly reduced water uptake of up to 90% after 600 hours of diffusion of water into the polymer test.

Predicting the lifespan of polymeric construction materials for the rapid prototyping and tool-making industry is another element that is required for ensuring an adequate product quality and reduced expenses. Musial et al. examined the micro- and macrostructures of seven samples (RenShape® series, Cibatool® series and phenolic cotton-laminated plastic laminate) before and after a test using a surface grinder for form and finish grinding [27]. They compared the abrasive wear resistance and surface roughness between the samples; however, more interestingly, based on the obtained results, they described the relationship between the material type and the wear resistance using several multiple regression equations in order to predict the wear resistance of the materials.

Another application of polymers is noise absorbers, because noise pollution is a negative environmental factor. Vasina and coworkers produced open-porous structured acrylonitrile butadiene styrene (ABS) materials with Cartesian, Starlit and Octagonal structures using 3D printing technology and measured their sound absorption properties by a transfer function method using an acoustic impedance tube [28]. The damping properties of the materials were evaluated in terms of a normal incidence sound absorption coefficient and the noise reduction coefficient, both in relation to various factors such as the open-porous material structure, the thickness of the sample, the excitation frequency and the gap size behind the sound-absorbing material inside the acoustic impedance tube. From the point of view of the open-porous material structure, the greatest noise attenuation capacity was observed in the Starlit structure when compared to the other ABS porous structures. Furthermore, the sound damping properties are generally low at low acoustic wave excitation frequencies, which are often eliminated by increasing the thickness of the material. However, the open-pore structure overcame this limitation, which allowed for the use of thinner materials, also resulting in a reduced cost and weight.

Developing technologies for appropriate respiratory monitoring is of great importance for various representative groups, such as newborns, people suffering from respiratory diseases or athletes. Danova and coworkers introduced a highly flexible piezoresistive strain sensor for breath detection [29]. Flexibility was ensured by a thermoplastic polyurethane, on which multiwalled carbon nanotubes (MWCNTs) capable of detecting deformation by changing the electrical resistance were deposited after electrospinning technology. In this study, the capacity of this breath-monitoring sensor was demonstrated by welding it to a prestrengthened condition and encapsulating it in a standard t-shirt.

Finally, Prof. Slobodian’s team developed new hybrid thermoelectric composites based on ethylene-octene-copolymer matrices containing conductive inorganic carbon and various conductive organic nanostructures, such as pure and functionalized MWCNTs, carbon nanofibers or the conductive polymers polyaniline and polypyrrole [30]. In this study, this composite system was investigated as a self-powered temperature change signal sensor and a self-powered vapor sensor represented by heptane vapors. Among other results, oxygen-doped MWCNTs were shown to contain a higher concentration of carboxyl and carbonyl functional groups on the surface of the MWCNTs, which increased the thermoelectric performance of the composite by up to 150% when compared to a system based on nonfunctionalized MWCNTs. The voltage induced in the sensor by the mentioned external stimuli was in microvolts, which was sufficient to make the sensor be self-powered.

## Data Availability

Data sharing not applicable.
